# CINPred: a risk prediction tool for cervical intraepithelial neoplasia

**DOI:** 10.3389/fonc.2026.1702579

**Published:** 2026-02-10

**Authors:** Jiaxuan Gu, Qiao Wang, Aili Li, Penghui Li, Saicong Lu, Zhen Wang, Lin Du, Feifei Zhao, Tingting Zhao, Feng Tian

**Affiliations:** 1 Hebei Key Laboratory of Medical Data Science, Institute of Biomedical Informatics, School of Medicine, Hebei University of Engineering, Handan, Hebei, China; 2 School of Information and Electrical Engineering, Hebei University of Engineering, Handan, Hebei, China; 3 Hipro Biotechnology CO., LTD, Shijiazhuang, Hebei, China; 4 Department of Gynecology, Handan Central Hospital, Handan, Hebei, China; 5 Department of Dermatology, Shanghai Ninth People's Hospital, Shanghai JiaoTong University School of Medicine, Shanghai, China

**Keywords:** CatBoost-based, cervical intraepithelial neoplasia, CINPred, early detection of cervical cancer, machine learning, SHAP

## Abstract

**Introduction:**

Cervical intraepithelial neoplasia (CIN) is a group of precancerous lesions associated with invasive carcinoma of the cervix that reflects the continuous progression of cervical cancer (CC). Therefore, early detection and standard treatment can effectively prevent the progression of CIN to CC. The objective of this study is to establish machine learning model using clinical data to predict the risk of CIN in women, and to develop a clinical prediction tool, exploring its broader clinical application significance.

**Methods:**

Female patients who sought consultation for cervical lesions at a hospital in Jiangsu province between 2018 and 2021 were enrolled in this study. The feature variables considered in the analysis included age, ThinPrep cytological test (TCT), human papillomavirus (HPV) genotype, multiple infection assessment, folate receptor-mediated tumor detection (FRD) and cotton-tipped swab test. Several algorithms were utilized for establishing the model, including adaptive boosting (AdaBoost), gradient boosting decision tree (GBDT), categorical boosting (CatBoost) and others. The performance of models was rigorously evaluated. The SHapley Additive exPlanation (SHAP) values were used to identify risk factors affecting the risk of CIN.

**Results:**

For predicting CIN events, CatBoost and GBDT had the highest area under the receiver operating characteristic curve (AUC) (0.89, 0.87, respectively). AdaBoost had the highest F1 score (F1 score = 0.81), followed by RF, LR and stochastic gradient descent (SGD). SHAP values suggested that the variables affected the risk of CIN in descending order of magnitude were TCT, age, FRD, cotton-tipped swab, multiple infection and HPV, respectively.

**Discussion:**

A novel CatBoost-based risk prediction tool for CIN (CINPred) has been developed and it can be accessed through the website at: https://medinfo.hebeu.edu.cn/shiny/CINPred/. CINPred can be used as a quick screening tool to assess CIN risk, offering significant benefits for the development of personalized treatment plans.

## 1 Introduction

Cervical cancer (CC) is the fourth most common cancer among women worldwide and is a global public health problem closely related to women’s health (1), with a particularly high burden in many low and middle income countries (LMICs) (2). According to a World Health Organization (WHO) survey in 2022 on CC, there were about 660,000 new cases and about 350,000 deaths. The incidence of CC has been high for a long time. The global strategy of the WHO CC Elimination Initiative (CCEI) is to reduce the incidence to a threshold of less than 4 cases per 100,000 women every year in this century, thereby eliminating the disease as a public health problem (3).

Cervical intraepithelial neoplasia (CIN) is a precancerous lesion that precedes invasive CC that reflects the continuum of cervical carcinogenesis (4). CIN is categorized into three grades: CIN 1, CIN 2 and CIN 3. Most CIN 1 cases can resolve naturally, while some CIN 2 and CIN 3 cases have the potential to develop into cancer (5). From HPV infection to cervical carcinogenesis is a long and reversible pathological process (6). Therefore, early screening to detect CIN and timely treatment are crucial in reducing both morbidity and mortality (7). TCT (ThinPrep cytological test) offers a cytomorphological basis for diagnosis, but TCT results are not only related to the clinician’s interpretation ability, but are also susceptible to false positives due to the sampling method (8). HPV testing has the advantages of fewer human factors and high detection rate. But it can only determine whether the patient has viral infection and HPV genotypes (9). FRD is easy to operate, but diagnostic errors caused by subjective interpretation cannot be entirely ruled out. These screening methods all have diagnostic value for CC, but each individual method has its limitations. Combining the three screening methods results in significantly enhanced diagnostic performance (10). Pathological tissue biopsy, as the gold standard for clinical diagnosis of CC, has a high accuracy rate. However, due to the need to take cervical tissue cells, it poses a risk of secondary damage caused by infection, is more expensive and requires a higher level of diagnostic expertise. It is unsuitable for large-scale screening (11). Therefore, it is essential to employ an auxiliary diagnostic tool to predict a patient’s risk level of CIN before undergoing a pathological tissue biopsy. The objective is to facilitate timely detection and treatment of those at high risk individuals (11), thereby reducing the unnecessary time and financial burden associated with patients traveling for biopsies, while enhancing the accuracy and cost-effectiveness of CC screening.

Machine learning (ML) has received much attention for its superior performance in disease risk prediction tools. Several studies on CC-related ML models based on public datasets have emerged. Mavra Mehmood et al. (12) proposed a method called “CervDetect” to assess the risk elements of malignant cervical formation based on 4 target parameters (biopsy, cytology, schiller and hinselmann) and 32 risk factors collected from the UCI CC data set, using random forest algorithm for feature selection important features followed by shallow neural network based detection of CC. Mengjie Chen et al. (10) included 120 cases in the Department of Gynecologic Oncology of the Affiliated Cancer Hospital of Guangxi Medical University in their study. Combining the clinical features and significantly differentially expressed genes of CIN patients, they explored the risk factors for the development and progression of CIN and established a multifactorial prediction model to predict the occurrence of CIN. Asadi F et al. (13) developed a study on 145 patients from Shohada Hospital in Tehran Iran from 2017 to 2018. They used decision tree to identify important characteristic variables (individual health level, marital status, social status, dose of contraceptive used, education level and number of cesarean sections) and applied support vector machine (SVM), QUEST, C&R tree, multilayer perceptron (MLP) and radial basis function (RBF) algorithms to successfully predict CC. The study based its predictions on socio-demographic characteristics and lacked validity and feasibility in a real clinical setting. The limitation of data quantity and the complexity of features make these models difficult to generalize.

Therefore, the aim of the present paper was to develop interpretable ML models based on relevant screening indicators from patients attending the cervical lesion clinic of a hospital in Jiangsu province, in order to accurately predict the risk of CIN at an early stage. The performance of each model was assessed objectively and comprehensively, with the importance of features clarified and the models interpreted using the SHapley Additive exPlanation (SHAP) method. Furthermore, we developed an online CIN risk prediction tool called CINPred and explored the practical applications of ML models in clinical practice to assist physicians in the screening of CC.

## 2 Methods

### 2.1 Study approval

This study was approved by the Biomedical Ethics Committee of School of Medicine, Hebei University of Engineering (no. BER-YXY-2024044). The study was conducted in accordance with the Declaration of Helsinki. The personal information of each participant was anonymized and deidentified at collection prior to analysis. The requirement for informed consent was therefore waived.

### 2.2 Study population

Participants were women who underwent cervical biopsy at a hospital in Jiangsu province between 2018 and 2022. The data is anonymized and there is no patient privacy involved. The data mainly included age, TCT, HPV, multiple infection, FRD, cotton-tipped swab and cervical pathological tissue biopsy results. Among these, pathological tissue biopsy was the outcome variable. Ultimately, 570 participants were recruited after applying the following inclusion criteria (1): women >= 18 years old & <= 100 years old (2); pathological tissue biopsy performed with complete and reliable results.

### 2.3 Data preprocessing

**Figure 1:**
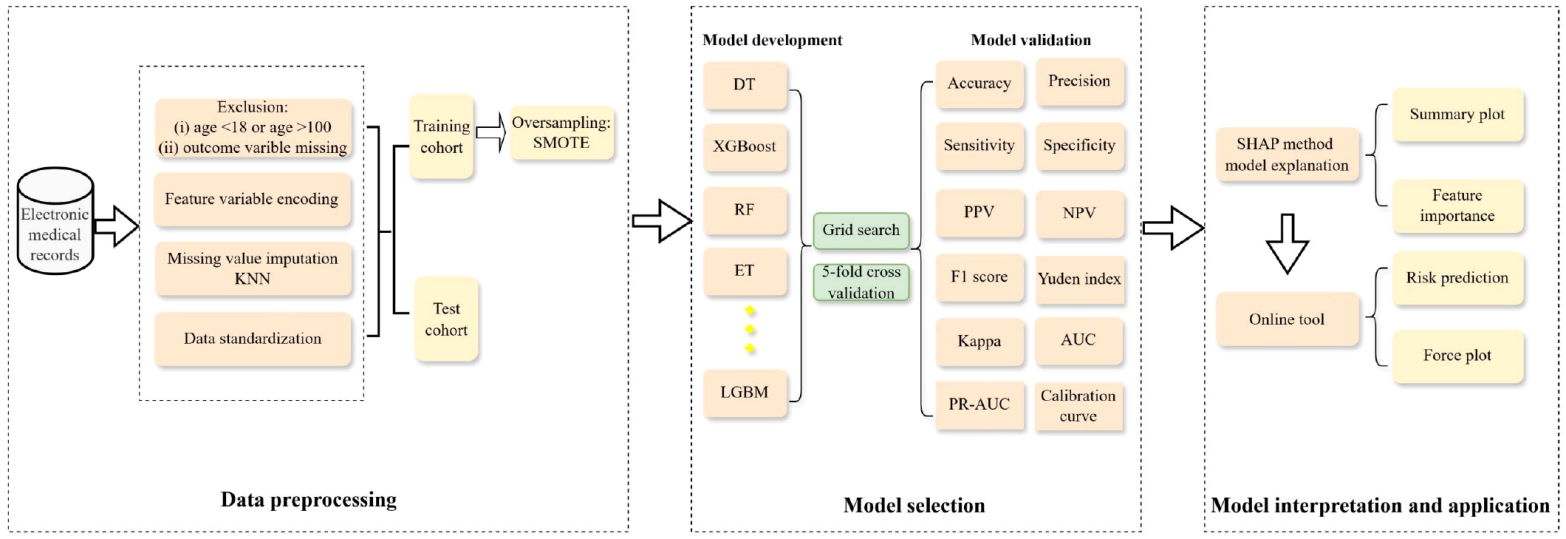
The flow diagram of data processing and model building process.

The original data set may contain problems such as missing values, outliers, or uneven sampling. Consequently, it is necessary to pre-process it to obtain high quality data. The analysis process was shown in **Figure 1**.

First, samples with missing outcome variables were removed. In order to enable ML models to process and interpret categorical features, non-numerical categorical labels were converted into numerical data (Label Encoding). Label Encoding maps each unique classification label to a unique integer by building a mapping dictionary. The coding results are listed in **Table 1**. The results of TCT were interpreted according to the TBS cervical cell classification (14): negative for intraepithelial lesions or malignancy (NILM), atypical squamous cells of undetermined significance (ASC-US), low-grade squamous intraepithelial lesion (LSIL), atypical squamous cells cannot exclude high-grade squamous intraepithelial lesion (ASC-H), high-grade squamous intraepithelial lesion (HSIL). HPV genotypes were classified based on their propensity to cause CC (15), including negative, high-risk HPV (HR-HPV) (HPV 16, 18, 31, 33, 35, 39, 45, 51, 52, 58, 59, 66 and 68) and low-risk HPV (LR-HPV) (positive genotypes other than HR-HPV) (16). The multiple infection assessments were classified as negative, single infection (people infected with one HPV genotype) and multiple infection (people infected with multiple HPV genotypes) (17). FRD was classified into two categories: no lesions (the swabs were brown, green or colourless), with intraepithelial neoplasia (the swabs were dark green, black or blue) (18). The results of cotton-tipped swab test were classified as negative, suspicious and positive. Based on the results reported of the histopathology report, CIN 2, CIN 3, squamous cell carcinoma (SCC), microinvasive carcinoma, adenocarcinoma *in situ*, adenocarcinoma (ACC) and CC were uniformly classified as CIN grade 2 or higher (CIN 2+). Chronic cervicitis, cervical polyp and CIN 1 were classified as CIN grade 2 or lower (CIN 2-) (19).

**Table 1:** Features description information.

Features	Type	Description
Age	Continuous	Age at examination
TCT	Categorical	0: NILM, 1: ASC-US, 2: LSIL, 3: ASC-H, 4: HSIL
HPV	Categorical	0: Negative, 1: LR-HPV, 2: HR-HPV
Multiple infection	Categorical	0: Negative, 1: Single infection, 2: Multiple infection
FRD	Categorical	0: No abnormal cervical lesions, 1: Abnormal cervical lesions
Cotton-tipped swab	Categorical	0: Negative, 1: Suspicious, 2: Positive

TCT (ThinPrep cytological test), HPV (human papillomavirus), FRD (folate receptor-mediated tumor detection), Multiple infection (the result of determining how many HPV genotypes (one or multiple) the patient is infected with), Cotton-tipped swab (the assessment outcome of the cotton-tipped swab test).

In this retrospective study, python (version 3.12.4) and KNNImputer of scikit-learn library were used for filling missing values. Due to the imbalance in the data set categories, the positive samples are oversampled on the training set using synthetic minority oversampling technique (SMOTE). It should be noted that only the training set was used to apply SMOTE, not the test set. SMOTE balances the classes of the data set by increasing the number of minority classes of K-nearest neighbors to near equal classes, bridging the gap between minorities and majorities (20). The process was performed using the imblearn library. Ultimately, data normalization was performed and the features were scaled.

### 2.4 Model development

Several algorithms were used to build the prediction model, including decision tree (DT), random forest (RF), logistic regression (LR), support vector machine (SVM), k-nearest neighbor (KNN), gradient boosting decision tree (GBDT), extreme gradient boosting (XGBoost), Gaussian naive Bayes (Gaussian NB), light gradient boosting machine (LGBM), categorical boosting (CatBoost), extremely randomized trees (ET), stochastic gradient descent (SGD), adaptive boosting (AdaBoost) and artificial neural network (ANN). These models were selected to represent diverse modeling paradigms, including linear, distance-based, tree-based, ensemble, and neural network approaches, thereby enabling a systematic comparison of predictive performance and robustness under different modeling assumptions. To further evaluate the stability of the models under different random stratification ratios (training set:test set), we conducted multiple comparative experiments. Fourteen machine learning algorithms were employed, with models trained and evaluated under randomly stratified training-to-test set ratios of 6:4, 7:3, 8:2, and 9:1. Feature selection in this study was guided by clinical relevance and practical applicability rather than by automated data-driven feature elimination methods. The included variables were predefined based on routinely available cervical cancer screening indicators and established clinical evidence, with the aim of enhancing model feasibility and interpretability in real-world screening settings. To assess the relevance of these features, univariate and multivariate logistic regression analyses were first conducted to evaluate their statistical associations with CIN risk. Subsequently, SHAP analysis was applied to quantify the contribution of each feature within the machine learning models, thereby providing an additional, model-based validation of feature importance rather than *post hoc* interpretation. A hyperparameter space containing a set of potential values for each parameter was developed in order to obtain the best parameters before building the final ML model. This approach aimed to incorporate different combinations of model parameters to obtain the best model. To mitigate the potential instability associated with a smaller test set, we implemented rigorous internal validation strategies, including 5-fold cross-validation on the training set for each parameter combination and comprehensive model evaluation using multiple performance metrics, ensuring that the selected model was robust against overfitting and variability within the available data. Following the cross-validation results, the hyperparameter set that yielded the best performance was chosen, with the highest area under the receiver operating characteristic curve (AUC) serving as the selection criterion. Subsequently, the entire training set was retrained to achieve the optimal results.

### 2.5 Model evaluation

To validate the performance of the prediction model, several evaluation criteria were employed, including accuracy, precision, sensitivity, specificity, positive predictive value (PPV), negative predictive value (NPV), F1 score, Yuden index, kappa, AUC, area under precision-recall curve (PR-AUC) and calibration curve. The best model for the target population was then identified by comparing the discriminatory and calibration validity of the best models derived from different algorithms. In this section, the various metrics used to evaluate the performance of ML models were outlined. Accuracy is the ratio of correctly predicted outcomes to the total number of samples. Precision is the probability of all samples predicted to be positive cases actually being positive cases. Sensitivity (recall) is the probability that a sample that is actually a positive case will be predicted to be a positive case. Specificity is the proportion of all negative case samples predicted correctly to all actual negative case samples. PPV is used to assess the proportion of all individuals tested positive who actually have the disease. NPV is used to assess the proportion of all individuals tested who have a negative test result who actually do not have the disease. F1 score is a game of precision and recall. Yuden index combines model sensitivity and specificity. Kappa is a statistic that measures the performance of a classifier. AUC is used to measure the classifier performance (21). Class imbalance often occurs in real datasets and it is more stable to use receiver operating characteristic (ROC) curve as a measure of classification (22). PR-AUC focuses on the relationship between precision and recall, and is particularly suitable for unbalanced datasets. Calibration curve is used to test the agreement between the probabilities predicted by the model and the frequency of actual events (23).

### 2.6 Model interpretation

Model interpretation helps us understand the process of model classification (24). SHAP provides a quantitative assessment of the contribution of each feature in the model to the prediction (25). After model evaluation, the best model was selected comprehensively and the marginal contribution of features was calculated based on SHAP to explain the model output and the results were visualized.

The global interpretation of SHAP provides consistent and precise attribution values for each feature within the model, thereby revealing associations between input features and prediction outcomes. A two-axis SHAP visualization was created by combining a bee swarm plot with a bar plot. Additionally, force plot for a single patient was generated, showing how each feature contributes to the model’s prediction of a specific patient outcome. In force plot, SHAP values are visualized as forces, where each feature value acts as a force that either increases or decreases the prediction. The prediction starts from a baseline, which is a constant that represents the model’s average prediction in the absence of any feature effects. Each attributed value is represented by an arrow, with positive values increasing the prediction and negative values decreasing it.

### 2.7 Statistical analysis

The basic characteristics of the preprocessed data were analyzed descriptively. The baseline characteristics of the study population were represented as median when they were continuous variables, and as frequency (percentage) when they were categorical variables. The differences in variables between CIN 2- group and CIN 2+ group were analyzed. The t test or Mann–Whitney test was used for continuous variables. The chi-square test or Fisher’s exact test were used for categorical variables. Statistical significance was inferred at a two-sided p value < 0.05.

Univariate logistic regression and multivariate logistic regression analyses were performed to assess risk factors for CIN. Statistical analysis was performed by R (version 4.2.1). P value < 0.05 was considered statistically significant.

### 2.8 Online tool

In order to enhance the value of the model for application in a clinical setting, a Web-based risk prediction tool was developed using shiny. When corresponding feature values in the model are specified, the server can generate both the CIN risk and the force plot for individual patients.

## 3 Results

### 3.1 Characteristics of participants

A total of 570 subjects were included in the study, of whom 268 (47.02%) were CIN 2+ patients and 302 (52.98%) were CIN 2- patients. **Table 2** depicts Baseline characteristics of the participants. The study population was divided into a training set (n=513) and a test set (n=57). Differences in TCT, HPV, multiple infection, FRD and cotton-tipped swab between the two groups were statistically significant (p value < 0.05) (**Table 3**). Characteristics in the training and test cohorts are shown in **Supplementary Tables 1, 2**.

**Table 2:** Baseline characteristics of the participants.

Characteristics	Total	CIN 2-	CIN 2+	P value
Age (median)	23-70 (43)	23-70 (44)	26-70 (42)	0.012
TCT				<0.001
NILM	30.0%	38.1%	20.9%	
ASC-US	33.7%	38.7%	28.0%	
LSIL	5.1%	1.0%	9.7%	
ASC-H	14.39%	15.9%	12.7%	
HSIL	8.95%	0.7%	18.3%	
HPV				<0.001
Negative	4.6%	7.0%	1.9%	
LR-HPV	3.9%	6.3%	1.1%	
HR-HPV	78.1%	75.4%	81.0%	
Multiple infection				0.021
Negative	4.56%	7.0%	1.9%	
Single infection	58.1%	159.3%	56.7%	
Multiple infection	23.9%	22.5%	25.4%	
FRD				<0.001
No abnormal cervical lesions	42.1%	53.0%	29.9%	
Abnormal cervical lesions	57.9%	47.0%	70.1%	
Cotton-tipped swab				<0.001
Negative	29.6%	44.0%	13.4%	
Suspicious	62.1%	52.0%	73.5%	
Positive	8.2%	4.0%	13.1%	

TCT (ThinPrep cytological test), HPV (human papillomavirus), FRD (folate receptor-mediated tumor detection), Multiple infection (the result of determining how many HPV genotypes (one or multiple) the patient is infected with), Cotton-tipped swab (the assessment result of the cotton-tipped swab).

**Table 3:** Characteristics of the training and test cohorts.

Characteristics	Training cohort	Test cohort	P value
Age (median)	23-70 (43)	26-67 (47)	0.183
TCT			<0.001
NILM	31.0%	21.1%	
ASC-US	33.5%	35.1%	
LSIL	5.1%	5.3%	
ASC-H	14.6%	12.3%	
HSIL	8.4%	14.0%	
HPV			<0.001
Negative	3.9%	10.5%	
LR-HPV	3.5%	0.7%	
HR-HPV	78.8%	71.9%	
Multiple infection			0.011
Negative	3.9%	10.5%	
Single infection	58.3%	56.1%	
Multiple infection	24.0%	22.8%	
FRD			<0.001
No abnormal cervical lesions	42.5%	38.6%	
Abnormal cervical lesions	57.5%	61.4%	
Cotton-tipped swab			<0.001
Negative	30.4%	22.8%	
Suspicious	61.6%	66.7%	
Positive	8.0%	10.5%	

TCT (ThinPrep cytological test), HPV (human papillomavirus), FRD (folate receptor-mediated tumor detection), Multiple infection (the result of determining how many HPV genotypes (one or multiple) the patient is infected with), Cotton-tipped swab (the assessment result of the cotton-tipped swab).

In univariate logistic regression analysis, all variables were statistically significant (p value < 0.05). Multivariate logistic regression incorporated variables that were statistically significant after univariate analysis. The results showed that age, TCT, HPV, multiple infection and cotton-tipped swab were independent risk factors for CIN 2+ (**Table 4**).

**Table 4:** Univariate and multivariate logistic regression analysis.

Characteristics	Univariate logistic regression analysis				Multivariate logistic regression analysis			
	β	Odds ratio	95%CI	P value	β	Odds ratio	95%CI	P value
Age	-0.021	0.980	(-0.036 - -0.005)	0.016	-0.027	0.973	(-0.046 - -0.009)	0.004
TCT	0.506	1.659	(0.368 - 0.656)	<0.001	0.483	1.621	(0.332 - 0.640)	<0.001
HPV	0.836	2.308	(0.422 - 1.310)	<0.001	0.908	2.480	(0.390 - 1.471)	<0.001
Multiple infection	0.404	1.498	(0.082 - 0.731)	0.014	0.054	1.055	(-0.368 - 0.476)	0.080
FRD	0.974	2.648	(0.631 - 1.323)	<0.001	-0.286	0.755	(-0.816 - 0.244)	0.297
Cotton-tipped swab	1.342	3.826	(1.009 - 1.693)	<0.001	1.381	3.981	(0.896 - 1.885)	<0.001

TCT (ThinPrep cytological test), HPV (human papillomavirus), FRD (folate receptor-mediated tumor detection), Multiple infection (the result of determining how many HPV genotypes (one or multiple) the patient is infected with), Cotton-tipped swab (the assessment result of the cotton-tipped swab).

### 3.2 Model development and evaluation

The process of developing the model is shown in **Figure 1**. To ensure optimal performance of each ML model, a grid search algorithm was used to optimize and tune the model parameters, 5-fold cross-validation was used to reduce the impact of overfitting on the model, and the parameters of the ML model were tuned to the extent allowed by the model to obtain the best results. The comprehensive performance of the predictive model in the training set was shown in **Supplementary Table 3**. The stability and generalization ability of models were verified in the test set (**Table 5**).

**Table 5:** Comprehensive performance of prediction models on the test cohort.

Model	AUC	Accuracy	Sensitivity	Specificity	PPV	NPV	Precision	F1 score	Yuden index	Kappa
LR	0.8465	0.7719	0.9130	0.6765	0.6563	0.9200	0.6563	0.7636	0.5895	0.5544
SVM	0.8159	0.6842	0.6842	0.6176	0.5806	0.8077	0.7161	0.6866	0.3019	0.3789
RF	0.8331	0.7895	0.7895	0.7353	0.6897	0.8929	0.8109	0.7914	0.5248	0.5804
ET	0.8388	0.7719	0.7826	0.7059	0.6429	0.8276	0.6429	0.7059	0.4884	0.4719
DT	0.7289	0.7018	0.7018	0.6471	0.6000	0.8148	0.7281	0.7043	0.3488	0.4095
XGBoost	0.8133	0.7544	0.7826	0.7352	0.6667	0.8333	0.6667	0.7200	0.5179	0.5037
KNN	0.7743	0.6842	0.7391	0.6471	0.5862	0.7857	0.5862	0.6538	0.3862	0.3706
AdaBoost	0.8792	0.7719	0.7719	0.7353	0.7097	0.9615	0.7097	0.8148	0.6918	0.6550
GBDT	0.8766	0.7368	0.7368	0.6176	0.6176	0.9130	0.7938	0.7368	0.3545	0.4926
LGBM	0.8139	0.7193	0.7193	0.6471	0.6129	0.8462	0.7520	0.7214	0.3664	0.4479
Gaussian NB	0.7967	0.6316	0.7391	0.5588	0.5313	0.7600	0.5313	0.6182	0.2980	0.2802
SGD	0.8646	0.7719	0.9130	0.6765	0.6563	0.9200	0.6563	0.7636	0.5895	0.5544
ANN	0.8517	0.7368	0.7368	0.6765	0.6333	0.8519	0.7637	0.7391	0.4133	0.4790
CatBoost	0.8913	0.7544	0.9130	0.6471	0.6364	0.9167	0.6364	0.7500	0.5601	0.5233

PPV, positive predictive value; NPV, negative predictive value; AUC, area under the curve; DT, decision tree; RF, random forest; LR, logistic regression; SVM, support vector machine; KNN, k-nearest neighbors; GBDT, gradient boosting decision tree; XGBoost, extreme gradient boosting; Gaussian NB, Gaussian naive Bayes; LGBM, light gradient boosting machine; CatBoost, categorical boosting; ET, extremely randomized trees classifier; SGD, stochastic gradient descent; AdaBoost, adaptive boosting; ANN, artificial neural network.

**Figure 2:**
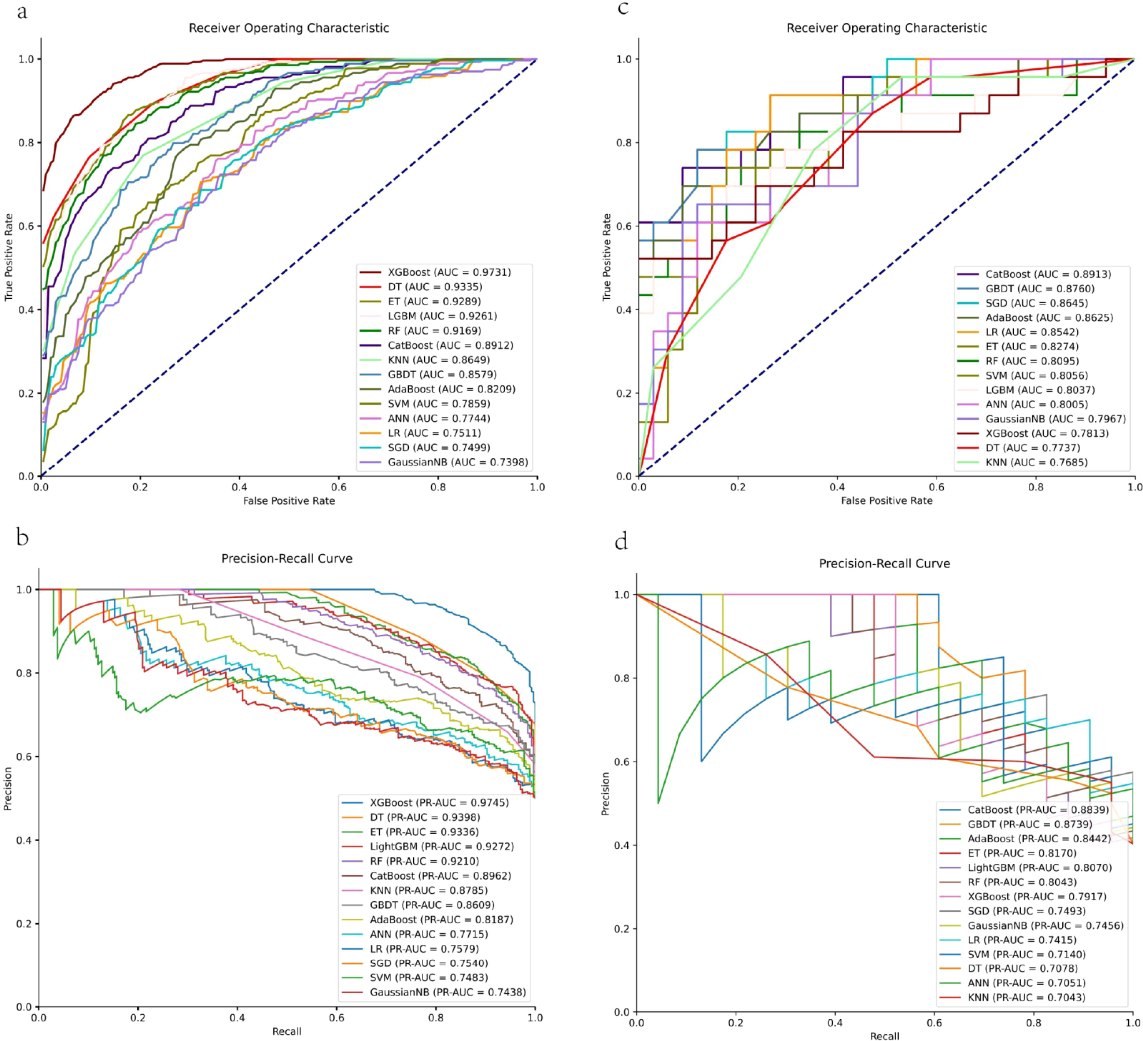
ROC curve and precision-recall curve. **(a)** ROC curve in the training set. **(b)** ROC curve in the test set. **(c)** PR curve in the training set. **(d)** PR curve in the test set. AUC, area under the curve; PR-AUC, area under precision-recall curve; DT, decision tree; RF, random forest; LR, logistic regression; SVM, support vector machine; KNN, k-nearest neighbors; GBDT, gradient boosting decision tree; XGBoost, extreme gradient boosting; Gaussian NB,Gaussian naive Bayes; LGBM, light gradient boosting machine; CatBoost, categorical boosting; ET, extremely randomized trees classifier; SGD, stochastic gradient descent; AdaBoost, adaptive boosting; ANN, artificial neural network.

We evaluated fourteen machine learning algorithms under randomly stratified training-to-test set ratios of 6:4, 7:3, 8:2, and 9:1. The results indicated that for the 6:4, 7:3, and 8:2 splits, the AUC values on the test sets were consistently lower than those obtained with the 9:1 split, and the performance gap between the training and test sets increased substantially (**Supplementary****Figures 1**-**3**). Specifically, for the 6:4 split, the training and test AUC values were 0.8613 and 0.8149, respectively; for the 7:3 split, the training AUC was 0.9033, whereas the test AUC decreased to 0.8202; for the 8:2 split, the training AUC reached 0.9259, while the test AUC dropped to 0.7731. These findings indicate that smaller training-to-test splits led to a pronounced increase in the discrepancy between training and test performance, reflecting reduced model stability and less reliable generalization. In contrast, the 9:1 split maintained sufficient training sample size and yielded highly consistent performance between the training and test sets, demonstrating optimal model stability and generalization capability. ROC curve and PR curve in the training set and test set was plotted (**Figure 2**). It was found that CatBoost had the highest AUC value (AUC = 0.8913), which was the best for predicting the CIN risk class, followed by GBDT (AUC = 0.8760), SGD (AUC = 0.8645) and AdaBoost (AUC = 0.8625). CatBoost exhibits excellent advantages in predicting the risk of CIN. Although the test cohort was limited in size (n=57), the model performance remained consistent with cross-validation results from the training set. For instance, the AUC of CatBoost in the test set (0.8913) closely aligned with the mean cross-validated AUC from the training phase (0.8912), indicating that the model generalizes reliably within the available data scope.

**Figure 3:**
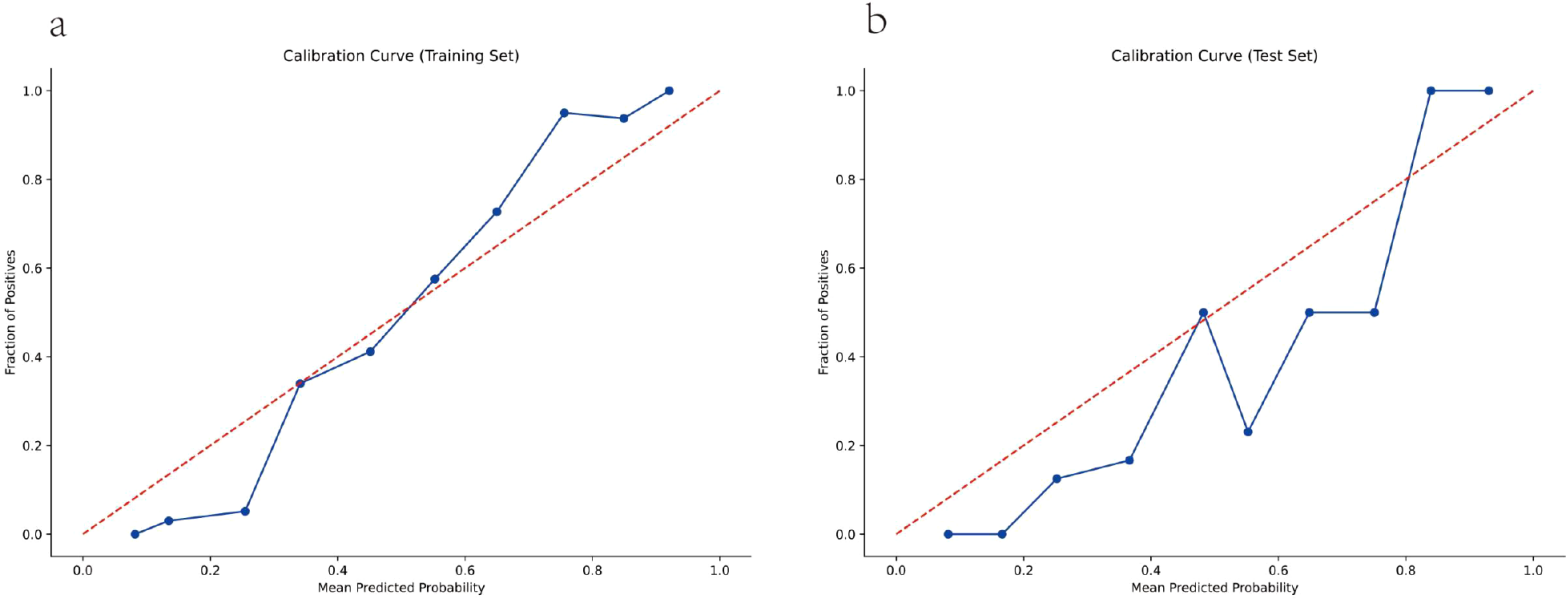
Calibration curve. **(a)** Training set. **(b)** Test set.

Calibration curve is used to assess predictive value. The calibration curve is close to the dotted line, indicating that the model’s predictions are highly consistent with the actual situation and the model has good calibration capability. Calibration curves revealed a good fit of the model for predicting CIN. The Brier scores were 0.161 and 0.173 in the training and test sets, respectively (**Figure 3**).

### 3.3 Model interpretation

**Figure 4:**
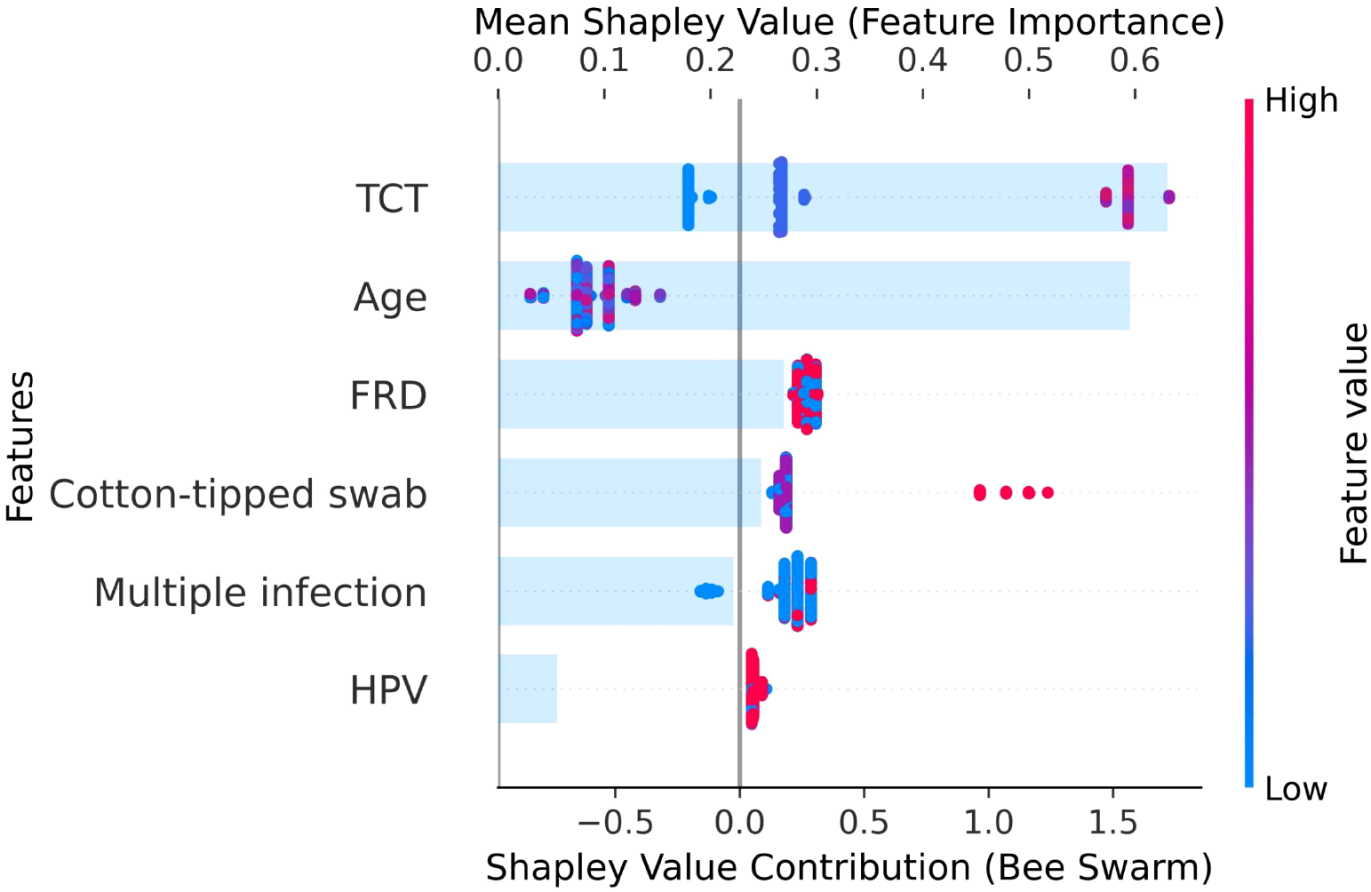
Dual-axis SHAP plot. TCT (ThinPrep cytological test), HPV (human papillomavirus), FRD (folate receptor-mediated tumor detection), Multiple infection (the result of determining how many HPV genotypes (one or multiple) the patient is infected with), Cotton-tipped swab (the assessment outcome of the cotton-tipped swab).

To elucidate the features contributions of model, SHAP values were utilized. **Figure 4** illustrates the extent to which each feature influences the CIN risk classification. Notably, the feature with the greatest impact on classification was TCT, followed by age, FRD, cotton-tipped swab, multiple infection and HPV, respectively.

### 3.4 Building of an online forecasting tool

**Figure 5:**
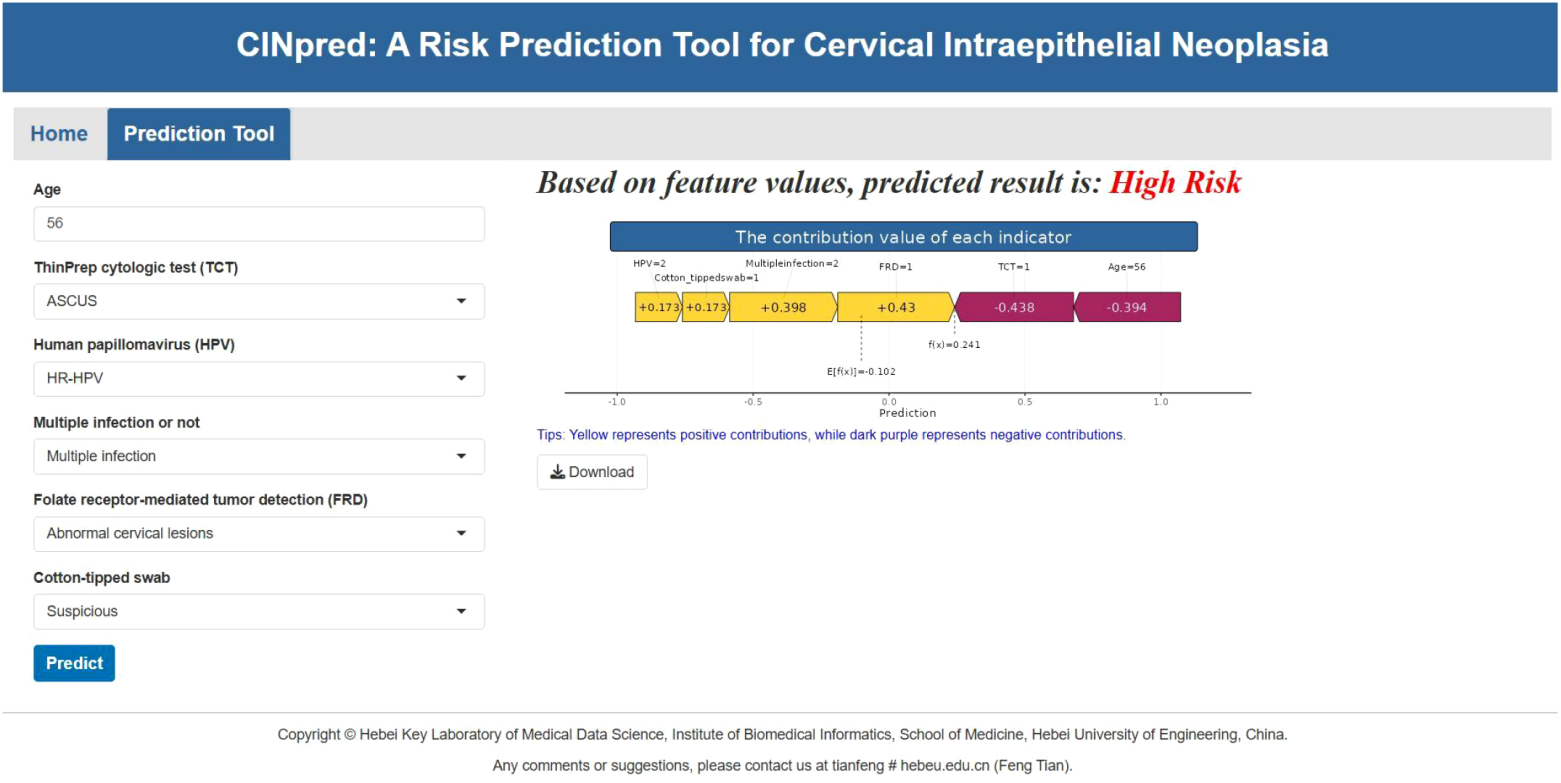
Online tool for predicting CIN.

As shown in **Figure 5**, CINPred was developed to facilitate the clinical application of the model. The application is available at https://medinfo.hebeu.edu.cn/shiny/CINPred/. It can predict the risk of CIN and display a force plot for an individual patient, which shows how each feature affects the model’s prediction of a specific patient outcome, adding transparency to the model’s decision-making process.

## 4 Discussion

Numerous studies have emphasized that CIN reflects the pathological process of cervical epithelium from abnormal proliferation to CC (26). The probability of CIN 1 and CIN 2–3 developing into invasive cancer of the cervix is 15% and 30-45% (27), respectively, which lasts for about 10 years. Early detection of CIN and targeted intervention can block the process of the lesion and reduce the probability of cancer (28). With the continuous accumulation of medical data, ML is widely used in the medical field (29). The development of disease classification prediction models is increasingly becoming a focal point and trend. Based on this, more than ten machine learning models were developed and validated to predict the risk of CIN using data from 597 clinical cases. Six key feature variables that significantly influenced CIN risk were identified and subsequently used as inputs for the machine learning models. CatBoost performed best (AUC = 0.89). CatBoost is an efficient gradient boosting algorithm developed by Yandex, which has significant advantages in dealing with categorization features (30). Using Shiny framework, CatBoost can be integrated into web pages and applied in clinical practice to assess the risk of CIN in individual patients, thereby informing improved screening, diagnosis, treatment and personalized interventions.

In addition, traditional interpretation methods of ML cannot adequately reveal the complex interactions between features and between features and predicted outcomes, which discourages physicians from making clinical decisions based on such opaque information in clinical applications. Therefore, SHAP was used to calculate the marginal contribution of features to interpret the output of the model (25). The dominant contribution of TCT results is consistent with current cervical cancer screening guidelines, reinforcing the central role of cytological findings in CIN risk stratification. Patients with abnormal TCT results were associated with higher predicted risks, suggesting that such individuals may benefit from closer surveillance or earlier referral for colposcopic examination. Age also showed a meaningful influence on risk prediction, reflecting the age-dependent distribution of cervical lesions. This finding indicates that age may serve as an important modifier when interpreting borderline or equivocal screening results, thereby supporting more refined, age-aware clinical decision-making. In addition, FRD and the use of cotton-tipped swab sampling emerged as relevant contributors in the SHAP analysis. Although these factors are not direct diagnostic indicators of CIN, their influence may reflect differences in sampling adequacy, specimen quality, or underlying inflammatory and anatomical conditions. From a clinical perspective, these findings highlight the potential value of procedural and sampling-related variables when interpreting screening results, particularly in resource-limited settings where rapid and low-cost indicators are essential. While HPV status and multiple infection showed relatively lower individual contributions, they provided complementary information when integrated with cytological and clinical features. This underscores the advantage of a multivariable risk prediction framework, such as CINPred, which reduces reliance on any single indicator and supports more balanced and individualized decision-making in primary cervical cancer screening.

Nonetheless, there are still some limitations to the current study. Since the clinical data collected by traditional methods cannot be used directly, they must be repeatedly calibrated and verified. Clinical data collection is more challenging. First, this study was conducted using a relatively moderate sample size (n = 570) collected from a single medical center in Jiangsu Province, and the external test cohort was relatively small (n = 57), which may limit the generalizability of the proposed model to broader populations. Although the dataset reflects real-world clinical practice and includes routinely used cervical screening indicators, potential selection bias and geographical constraints cannot be completely excluded. To enhance model robustness under these conditions, we employed stratified data splitting, five-fold cross-validation, comprehensive hyperparameter optimization, and independent test set evaluation. The model demonstrated consistent discrimination and calibration across internal and external sets, suggesting acceptable generalization within the target population. Nevertheless, future studies incorporating larger sample sizes, multicenter cohorts, and more diverse demographic characteristics are warranted to further validate and extend the applicability of the CINPred model. Second, the dataset utilized in this study did not include certain demographic and behavioral variables that may be associated with the risk of CIN, such as socioeconomic status, smoking behavior, sexual behavior characteristics, and prior medical history. These factors have been recognized in previous research as potentially influencing the onset and progression of cervical lesions, and their absence may, to some extent, limit further improvement in the model’s predictive performance. However, the primary objective of this study was to develop a CIN risk prediction model based on routine clinical screening indicators, emphasizing high operability and clinical utility. The selected features were all derived from standard clinical examination procedures, which are easily accessible and offer strong objectivity. This approach avoids potential reporting biases associated with self-reported demographic and behavioral information, thereby enhancing the feasibility of the model in real-world clinical screening settings. Future studies could build upon this work by incorporating additional information on demographics, lifestyle factors, and medical history to further refine model performance and expand its applicability. Third, although CINPred has been developed in this study, its validation has thus far been primarily based on retrospective data analysis. The tool has not yet been prospectively evaluated within real-world clinical screening workflows, nor has systematic feedback from healthcare professionals been formally collected. As a result, its practical usability, workflow integration, and clinical decision-support value in routine practice remain to be further assessed. Future work will focus on conducting prospective, multicenter clinical validation studies and incorporating feedback from gynecologists and related healthcare professionals to further optimize the tool’s functionality, risk stratification strategy, and real-world applicability.

## 5 Conclusion

The present study explored explainable models for predicting the risk of CIN by using patients’ clinical diagnostic indicators, enriching the field of prediction of cervical precancerous lesion risk based on clinical indicators. Furthermore, a prediction tool called CINPred was developed and it can be accessed through website at: https://medinfo.hebeu.edu.cn/shiny/CINPred/. It provides a practical tool for screening subjects with a potential risk of CIN.

## Data Availability

The data analyzed in this study is subject to the following licenses/restrictions: The datasets used and analyzed during the current study are available from the corresponding author on reasonable request. Requests to access these datasets should be directed to tianfeng@hebeu.edu.cn.
