# Supplementary material for: CINPred: a risk prediction tool for cervical intraepithelial neoplasia
Source: Front Oncol. 2026 Feb 10;16:1702579. doi: 10.3389/fonc.2026.1702579 (PMC12930185; doi:10.3389/fonc.2026.1702579)
Supplement: Supplementary file 1 [file DataSheet1.docx]

Supplementary Material

**Supplementary Table 1.** Characteristics in the training cohort

| **Features** | **Total** | **CIN 2-** | **CIN 2+** | **P value** |
| --- | --- | --- | --- | --- |
| Age(median) | 23-70 (43) | 23-70 (44) | 26-70 (42) | 0.018 |
| TCT |  |  |  | <0.001 |
| NILM | 31.0% | 39.2% | 22.0% |  |
| ASC-US | 33.5% | 38.1% | 28.6% |  |
| LSIL | 5.1% | 0.7% | 9.8% |  |
| ASC-H | 14.6% | 16.0% | 13.1% |  |
| HSIL | 8.4% | 0.7% | 16.7% |  |
| HPV |  |  |  | 0.001 |
| Negative | 3.9% | 6.0% | 1.6% |  |
| LR-HPV | 3.5% | 5.6% | 1.2% |  |
| HR-HPV | 78.8% | 76.5% | 81.2% |  |
| Multiple Infection |  |  |  | 0.030 |
| Negative | 3.9% | 6.0% | 1.6% |  |
| Single infection | 58.3% | 60.4% | 55.9% |  |
| Multiple infection | 24.0% | 21.6% | 26.5% |  |
| FRD |  |  |  | <0.001 |
| No abnormal cervical lesions | 42.5% | 54.9% | 30.0% |  |
| Abnormal cervical lesions | 57.5% | 45.1% | 71.0% |  |
| Cotton-tipped swab |  |  |  | <0.001 |
| Negative | 30.4% | 45.5% | 13.9% |  |
| Suspicious | 61.6% | 50.7% | 73.5% |  |
| Positive | 8.0% | 3.7% | 12.7% |  |

TCT (ThinPrep cytological test), HPV (human papillomavirus), FRD (folate receptor-mediated tumor detection), Multiple infection (the result of determining how many HPV genotypes (one or multiple) the patient is infected with), Cotton-tipped swab (the assessment result of the cotton-tipped swab).

**Supplementary Table 2.** Characteristics in the validation cohort

| **Features** | **Total** | **CIN 2-** | **CIN 2+** | **P value** |
| --- | --- | --- | --- | --- |
| Age(median) | 26-67 (47) | 26-67 (47) | 28-57 (42) | 0.463 |
| TCT |  |  |  | <0.001 |
| NILM | 21.1% | 29.4% | 8.7% |  |
| ASC-US | 35.1% | 44.1% | 21.7% |  |
| LSIL | 5.3% | 2.9% | 8.7% |  |
| ASC-H | 12.3% | 14.7% | 8.7% |  |
| HSIL | 14.0% | 0 | 34.8% |  |
| HPV |  |  |  | 0.247 |
| Negative | 10.5% | 14.7% | 4.3% |  |
| LR-HPV | 0.7% | 11.8% | 0 |  |
| HR-HPV | 71.9% | 67.6% | 78.3% |  |
| Multiple Infection |  |  |  | 0.299 |
| Negative | 10.5% | 14.7% | 4.3% |  |
| Single infection | 56.1% | 50.0% | 65.2% |  |
| Multiple infection | 22.8% | 29.4% | 13.0% |  |
| FRD |  |  |  | 1.000 |
| No abnormal cervical lesions | 38.6% | 38.2% | 39.1% |  |
| Abnormal cervical lesions | 61.4% | 61.8% | 60.9% |  |
| Cotton-tipped swab |  |  |  | 0.067 |
| Negative | 22.8% | 32.3% | 8.7% |  |
| Suspicious | 66.7% | 61.8% | 73.9% |  |
| Positive | 10.5% | 5.9% | 17.4% |  |

TCT (ThinPrep cytological test), HPV (human papillomavirus), FRD (folate receptor-mediated tumor detection), Multiple infection (the result of determining how many HPV genotypes (one or multiple) the patient is infected with), Cotton-tipped swab (the assessment result of the cotton-tipped swab).

**Supplementary Table 3.** Comprehensive performance of prediction models on the training cohort

| **Model** | **AUC** | **Accuracy** | **Sensitivity** | **Specificity** | **PPV** | **NPV** | **Precision** | **F1 score** | **Yuden index** | **Kappa** |
| --- | --- | --- | --- | --- | --- | --- | --- | --- | --- | --- |
| LR | 0.7511 | 0.6866 | 0.7276 | 0.6455 | 0.6724 | 0.7033 | 0.6724 | 0.6989 | 0.3731 | 0.3834 |
| SVM | 0.7859 | 0.7369 | 0.8209 | 0.6530 | 0.7029 | 0.7848 | 0.7029 | 0.7573 | 0.4739 | 0.4739 |
| RF | 0.9169 | 0.8172 | 0.8284 | 0.8060 | 0.8102 | 0.8244 | 0.8102 | 0.8148 | 0.6343 | 0.6194 |
| ET | 0.9289 | 0.8246 | 0.8396 | 0.8097 | 0.8152 | 0.8346 | 0.8152 | 0.8272 | 0.6493 | 0.5964 |
| DT | 0.9335 | 0.8806 | 0.8470 | 0.9142 | 0.9170 | 0.8407 | 0.9080 | 0.8764 | 0.7500 | 0.7612 |
| XGBoost | 0.9731 | 0.8675 | 0.8433 | 0.8918 | 0.8863 | 0.8505 | 0.8863 | 0.8642 | 0.7351 | 0.7351 |
| KNN | 0.8649 | 0.8190 | 0.8060 | 0.8321 | 0.8276 | 0.8109 | 0.8276 | 0.8166 | 0.6381 | 0.6574 |
| AdaBoost | 0.8209 | 0.7369 | 0.7351 | 0.7388 | 0.7378 | 0.7361 | 0.7378 | 0.7364 | 0.4739 | 0.4739 |
| GBDT | 0.8579 | 0.8451 | 0.8396 | 0.7090 | 0.7426 | 0.8155 | 0.7426 | 0.7881 | 0.5485 | 0.5734 |
| LGBM | 0.9261 | 0.7743 | 0.8246 | 0.8321 | 0.8333 | 0.8390 | 0.8308 | 0.8277 | 0.6567 | 0.6289 |
| Gaussian NB | 0.7398 | 0.6716 | 0.7910 | 0.5522 | 0.6386 | 0.7255 | 0.6386 | 0.7067 | 0.4985 | 0.3433 |
| SGD | 0.7499 | 0.6978 | 0.7164 | 0.6791 | 0.6906 | 0.7054 | 0.6906 | 0.7033 | 0.4352 | 0.3955 |
| ANN | 0.7744 | 0.7015 | 0.7015 | 0.6828 | 0.6942 | 0.7093 | 0.7018 | 0.7014 | 0.3843 | 0.4030 |
| CatBoost | 0.8912 | 0.7854 | 0.8433 | 0.7276 | 0.7559 | 0.8228 | 0.7559 | 0.7972 | 0.5937 | 0.5709 |

PPV, positive predictive value; NPV, negative predictive value; AUC, area under the curve; DT, decision tree; RF, random forest; LR, logistic regression; SVM, support vector machine; KNN, k-nearest neighbors; GBDT, gradient boosting decision tree; XGBoost, extreme gradient boosting; Gaussian NB, Gaussian naive Bayes; LGBM, light gradient boosting machine; CatBoost, categorical boosting; ET, extremely randomized trees classifier; SGD, stochastic gradient descent; AdaBoost, adaptive boosting; ANN, artificial neural network.


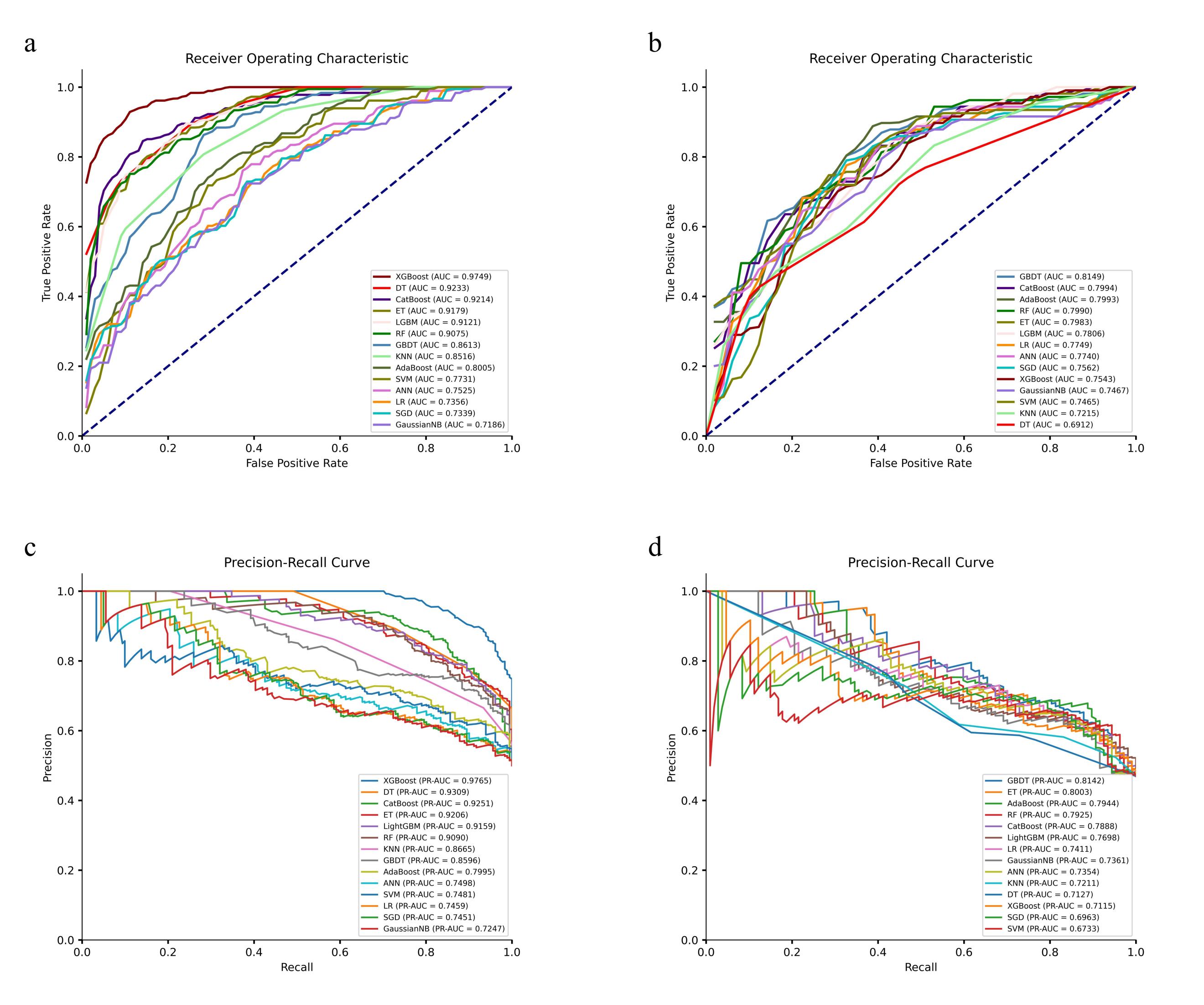


**Supplementary Figure 1.** ROC curve and Precision-recall curve on the 6:4 Train-Test split dataset.

Abbreviations: a ROC curve in the training set. b ROC curve in the test set. c PR curve in the training set. d PR curve in the test set. AUC, area under the curve; PR-AUC, area under precision-recall curve; DT, decision tree; RF, random forest; LR, logistic regression; SVM, support vector machine; KNN, k-nearest neighbors; GBDT, gradient boosting decision tree; XGBoost, extreme gradient boosting; Gaussian NB ,Gaussian naive Bayes; LGBM, light gradient boosting machine; CatBoost, categorical boosting; ET, extremely randomized trees classifier; SGD, stochastic gradient descent; AdaBoost, adaptive boosting; ANN, artificial neural network.


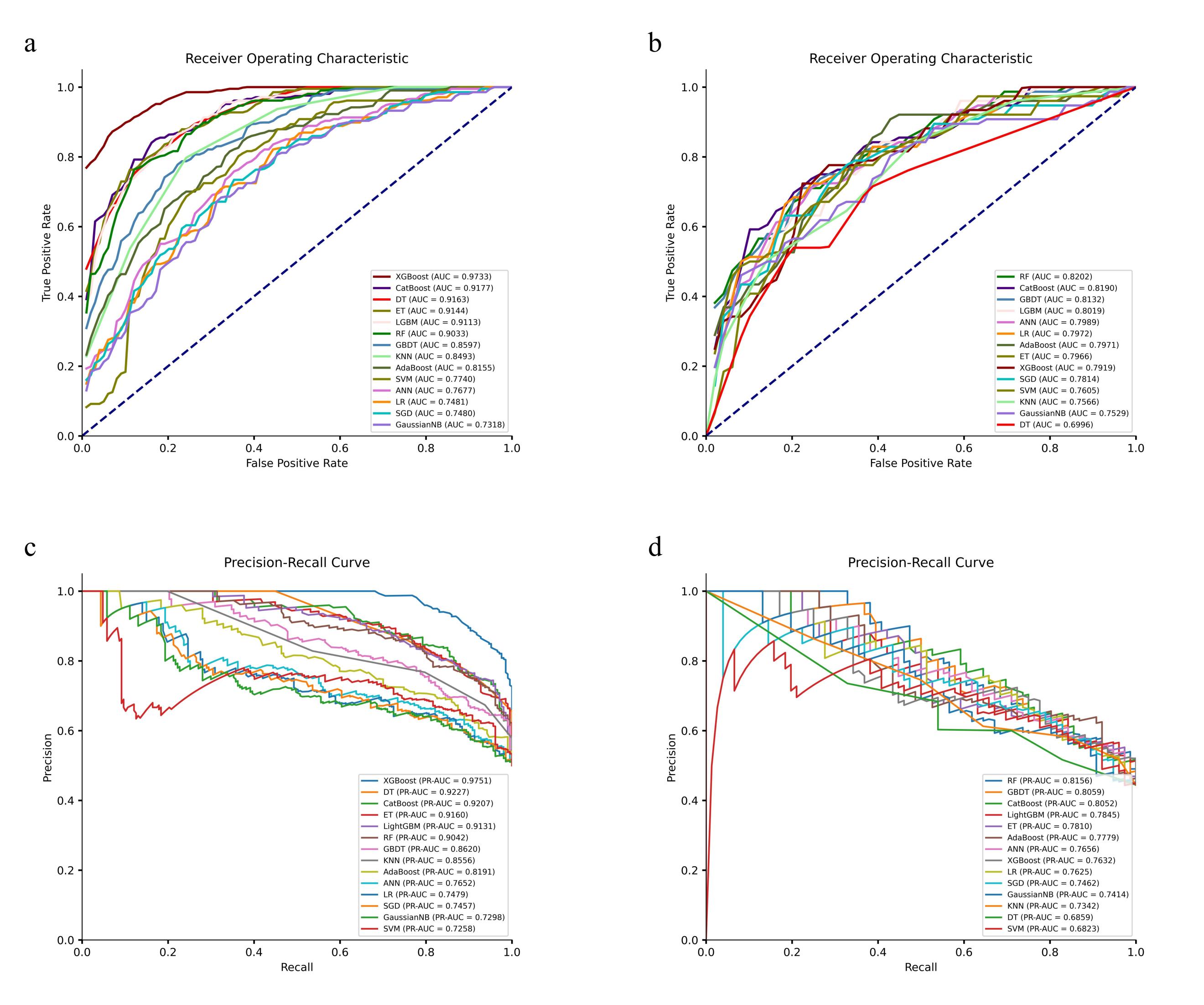


**Supplementary Figure 2.** ROC curve and Precision-recall curve on the 7:3 Train-Test split dataset.

Abbreviations: a ROC curve in the training set. b ROC curve in the test set. c PR curve in the training set. d PR curve in the test set. AUC, area under the curve; PR-AUC, area under precision-recall curve; DT, decision tree; RF, random forest; LR, logistic regression; SVM, support vector machine; KNN, k-nearest neighbors; GBDT, gradient boosting decision tree; XGBoost, extreme gradient boosting; Gaussian NB ,Gaussian naive Bayes; LGBM, light gradient boosting machine; CatBoost, categorical boosting; ET, extremely randomized trees classifier; SGD, stochastic gradient descent; AdaBoost, adaptive boosting; ANN, artificial neural network.


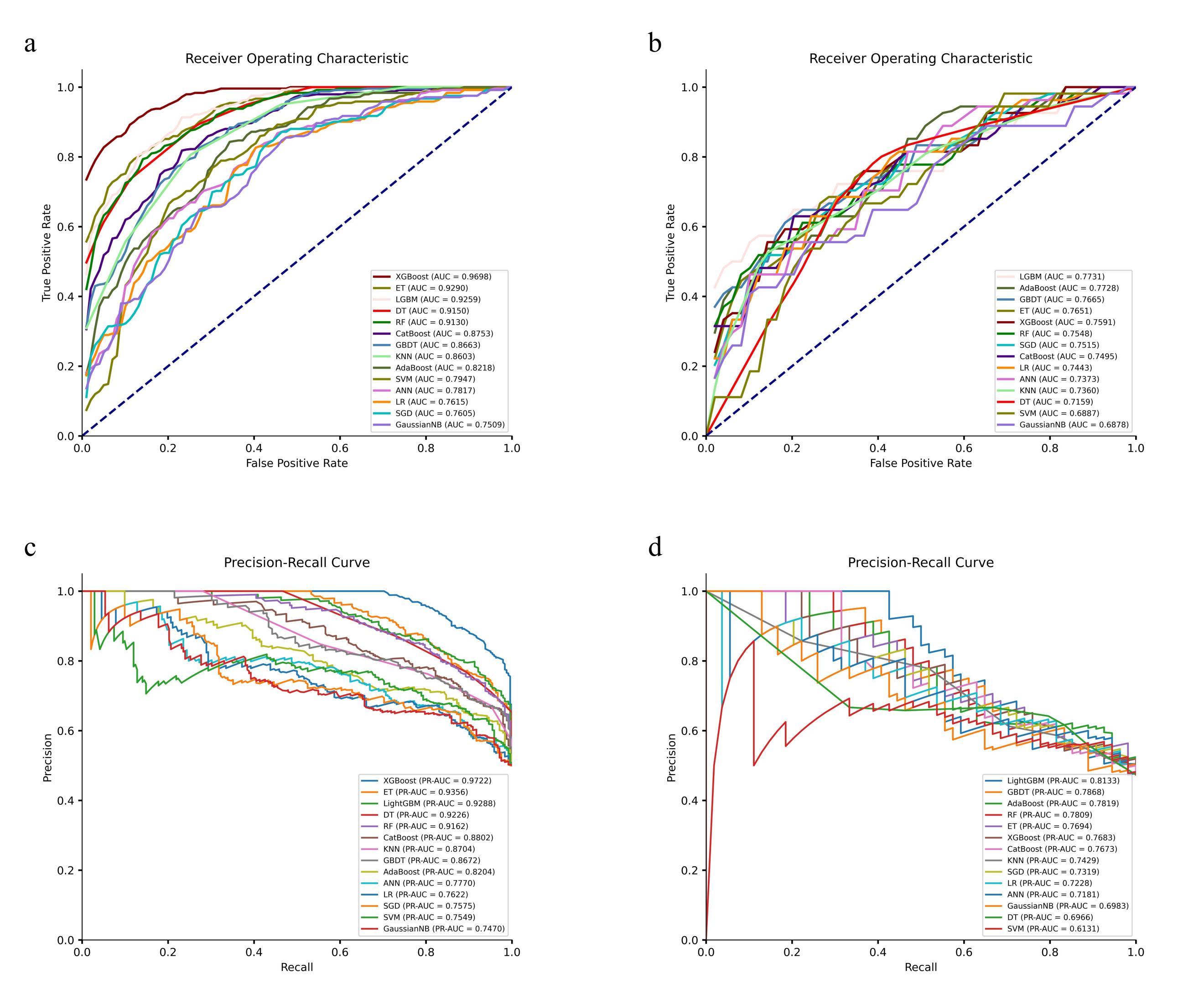
**Supplementary Figure 3.** ROC curve and Precision-recall curve on the 8:2 Train-Test split dataset.

Abbreviations: a ROC curve in the training set. b ROC curve in the test set. c PR curve in the training set. d PR curve in the test set. AUC, area under the curve; PR-AUC, area under precision-recall curve; DT, decision tree; RF, random forest; LR, logistic regression; SVM, support vector machine; KNN, k-nearest neighbors; GBDT, gradient boosting decision tree; XGBoost, extreme gradient boosting; Gaussian NB ,Gaussian naive Bayes; LGBM, light gradient boosting machine; CatBoost, categorical boosting; ET, extremely randomized trees classifier; SGD, stochastic gradient descent; AdaBoost, adaptive boosting; ANN, artificial neural network.
